# Co-designing a recruitment strategy for lung cancer screening in high-risk individuals: protocol for a mixed-methods study

**DOI:** 10.12688/hrbopenres.13793.2

**Published:** 2025-07-28

**Authors:** Brona Mulligan, Maeve Reilly, Ahmeda Ali, Conor Murphy, Sam McGlynn, Mohammed Alam, Frank Doyle, Seamus Cotter, Laura Heavey, Kate Brain, Nicole Rankin, Grace McCutchan, Patrick Redmond

**Affiliations:** 1 Department of General Practice, Royal College of Surgeons Ireland, Dublin, Ireland; 2 Department of Health Psychology, School of Population Health, Royal College of Surgeons Ireland, Dublin, Ireland; 3 Patient and Public Involvement (PPI), Irish Lung Cancer Community, Dublin, Ireland; 4 National Screening Service, Health Service Executive (HSE), Dublin, Ireland; 5 Division of Population Medicine, Cardiff University, Cardiff, UK; 6 School of Population and Global Health, The University of Melbourne, Melbourne, Victoria, Australia

**Keywords:** Lung neoplasms; Primary Health Care; Screening; Implementation Science; Health Knowledge, Attitudes, Practice

## Abstract

**Background:**

Lung cancer is a significant cause of cancer-related mortality globally, with early detection through screening critical to improving patient outcomes. However, recruiting high-risk individuals, particularly in deprived populations, for screening remains a considerable challenge. This study aims to co-design a targeted recruitment strategy for lung cancer screening, tailored to the specific needs and experiences of high-risk individuals, in collaboration with a Patient and Public Involvement (PPI) panel and expert stakeholders in Ireland.

**Methods:**

We will employ a mixed-methods design guided by the Medical Research Council (MRC) framework for developing complex interventions. Our approach will integrate systematic review findings on screening participation interventions, evaluation of the recruitment strategy's feasibility in an Irish context, and the application of behavioural science frameworks. The target population includes individuals aged 55–74 who reside in highly deprived areas and are at high risk of developing lung cancer based on the National Lung Screening Trial criteria, as well as those eligible for our Lung Health Check trial who met equivalent risk thresholds.

**Conclusion:**

This co-designed recruitment strategy will combine evidence-based research, local context understanding, and stakeholder input to develop a solution that is both scientifically robust and tailored to the target population's needs. This patient-centred approach aims to increase the potential for successful implementation of lung cancer screening programs, thereby improving early detection and patient outcomes.

## Introduction

Lung cancer is a significant global health concern, accounting for a substantial proportion of cancer-related mortality worldwide ^1^. Despite advances in treatment options, the overall prognosis for patients with lung cancer remains poor, primarily due to late-stage diagnosis ^2^. Emphasising the importance of early detection, screening programs have been identified as a key factor in improving patient outcomes, including overall survival rates ^3^.

### Challenges in lung cancer screening programmes

Screening programs, while instrumental in early detection, come with their own set of challenges. A major hurdle lies in the recruitment of eligible individuals, particularly those classified as high risk. Previous trials employing population-level approaches to recruitment ^4, 5^ have suffered from low participation rates. Underrepresentation of high-risk participants and participants from low socioeconomic (SES) background have been a critical challenge to lung cancer screening ^6– 8^. In contrast, the Lung Screen Uptake Trial (LSUT) and the Manchester Lung Health Check both used a targeted recruitment strategy, resulting in participation rates of 53% and 27% respectively ^9, 10^. Although the Manchester Lung Health Check participation rate may be comparable to other trials, it is worth noting that the proportion of participants ranked in the lowest deprivation quintile was 75%, a marked increase from the overall UK Lung Screen trial ^7, 10^.

Evidence regarding the impact of specific recruitment strategies on participation in cancer screening trials is limited. For examples, in the LSUT study, a reminder letter with a pre-scheduled appointment increased participation among non-responders by 24% ^9^. Similar strategies such as pre-invitation letters, scheduled appointments, and reminder letters have modestly increased participation in breast and colorectal cancer screening, albeit with varying success rates ^11– 14^. These studies demonstrate the impact that specific recruitment strategies can add to baseline recruitment rates, but they were not targeted to a specific population.

### Understanding high-risk individuals

Due in part to the prevalence of smoking in deprived areas, individuals from socioeconomically disadvantaged backgrounds are often at a higher risk of developing lung cancer ^15– 17^. This demographic is consistently underrepresented in screening programs ^18^, leading to disparities in lung cancer outcomes, with socioeconomically disadvantaged groups experiencing higher mortality rates ^19^.

Barriers to screening participation are multifaceted and complex, as suggested by various systematic and literature reviews ^20– 22^. According to Lin *et al.*, these barriers can be categorised into individual, healthcare system, and social/environmental categories ^22^. Several potentially modifiable factors have been identified, which could be strategically addressed in recruitment interventions. These encompass health beliefs, desires to quit smoking, self-efficacy beliefs, concerns about lung cancer, and prompts or recommendations from healthcare professionals ^22^. Moreover, Lin *et al.* and Lam *et al.* underscore the role of psychological factors, including apprehension and fatalistic attitudes, as barriers to screening uptake. Schütte *et al.* further highlights an inverse correlation between screening uptake and poor self-reported health ^20– 22^. As such, efforts to alleviate these anxieties could complement strategies designed to foster engagement and awareness, subsequently encouraging participation among these high-risk individuals.

### Tailored recruitment strategies

Addressing these challenges requires a targeted recruitment strategy, grounded in evidence-based practices and tailored to the unique needs and preferences of high-risk groups within an Irish context. Previous strategies aimed at increasing screening attendance have been primarily invitational or proactive. The LSUT trial and the Lung Health Check trials in the UK used invitational strategies, sending letter invitations, reminders and, in the LSUT, pamphlets with low-burden information about screening through direct mail ^9, 10^. The UK Bowel Cancer Screening programme trialled four different invitational strategies to decrease the socioeconomic gradient between screening participants and found enhanced reminder letters to have a modest benefit ^23^.

Proactive recruitment strategies have been more commonly trialled in non-lung screening programmes such as cervical, breast and colorectal cancer screening. Strategies such as flagging patient files to encourage their general practitioners to discuss screening with them or sending invitations with pre-scheduled appointments have worked to enhance general participation ^24, 25^. At least two lung cancer screening trials have effectively assigned participant “navigators” to guide participants through the screening process; from calling to invite participants and checking their eligibility to booking their appointments ^26, 27^.

A systematic review of lung cancer screening implementation supports the view that recruitment should be targeted to the most high-risk population and that the methodology for achieving this must be tailored to the local circumstance ^28^. However, the review also highlights that there is currently insufficient evidence to show which methods will best enhance screening uptake, and that more research into planning recruitment strategies is needed to identify how best to attract high-risk populations ^28^. In response to these identified gaps, this study aims to develop a recruitment strategy specifically designed for high-risk individuals within the Irish context. We hypothesize that integrating multiple recruitment modalities will maximize participation and engagement in lung cancer screening. Through co-design and rigorous evaluation, this study aims to identify the most effective approaches for reaching and engaging these populations.

### Patient and Public Involvement

Engaging patients and the public in the design and implementation of research ensures interventions are relevant, acceptable, and effective for the target population. In the context of lung cancer screening, patients can provide invaluable insights into participation barriers and potential solutions ^29^. Hence, this study will incorporate a Patient and Public Involvement group to offer insights into the feasibility and compatibility of the recruitment strategy within the local healthcare context ^30, 31^.

### Aims and objectives

The overall aim of this study is to co-design an evidence-based recruitment strategy that enhances participation in lung cancer screening among high-risk individuals. This strategy will be specifically tailored to the unique needs and experiences of high-risk individuals in Ireland. This study is being conducted in parallel with the LHC pilot trial, which is operating in regions with relatively high deprivation and lung cancer incidence. 

To achieve this we have established the following specific objectives:

**Evidence synthesis:** We will draw from the knowledge gathered through systematic reviews and observational studies to identify interventions that have demonstrated effectiveness in enhancing screening participation ^19, 23, 32– 34^.

**Addressing barriers:** In collaboration with high-risk individuals eligible for screening, we will identify and address the barriers to participation that are unique to the Irish context. This approach will ensure the recruitment strategy is practical and directly relevant to the target population.

**Theoretical grounding:** We will ground the recruitment strategy in behavioural science frameworks, which will provide a theoretical basis for understanding health behaviours. This will guide the design of interventions aimed at altering these behaviours to improve screening participation ^35^

**Feasibility assessment:** We will assess the feasibility of the recruitment strategy in the Irish context, employing the APEASE Criteria. This evaluation framework will ensure the intervention is affordable, practical, effective, acceptable, safe, and equitable ^36^.

## Methods

### Ethics

Ethical approval will be requested from RCSI Research Ethics Committee. We have routine systems in place to offer assistance and follow-up any patient who is distressed by health-related research. This includes signposting opportunities for support plus personal follow-up at participant request.

### Patient and Public Involvement

Irish Lung Cancer Community (ILCC)

### Framework and reporting guidance

This research employs a mixed-methods design with the aim of co-creating a targeted recruitment strategy for lung cancer screening, in collaboration with a PPI panel and expert stakeholders. By embedding this work within the ongoing LHC trial, we aim to generate actionable, context-specific insights that can inform the design of more equitable lung cancer screening programmes. The development and evaluation of the intervention will be guided by the Medical Research Council's (MRC) framework for complex interventions ^30^. The study will also adhere to guidelines for formulating complex interventions to enhance health and healthcare ^30^, and will follow the GUIDED reporting guidance for intervention development studies in health research ^37^. In accordance with the recommendations from a recent multidisciplinary expert panel review, the study will incorporate co-design methods using an Experience Based Co-Design approach ^38^.

Research-refining interviews with screening-eligible participants will be conducted to prioritise the issues specific to an Irish context. Intervention co-development workshops with expert and PPI participants will be conducted to build upon existing research evidence. These workshops will generate iterative versions of a recruitment tool until consensus on a final tool is reached ^39, 40^. The feasibility and acceptability of the recruitment tool will also be evaluated at these workshops. The Consolidated Framework for Implementation Research (CFIR) will be employed to assess factors likely to impact the intervention’s implementation and effectiveness. The RE-AIM framework will be used to enhance the external validity of the project and to orientate the data collection in a manner conducive to the future implementation of the final intervention ^41^.

### Research team and context

The research team is interdisciplinary, comprising individuals with diverse expertise: patients, academics from various disciplines (public health, epidemiology, health psychology, social sciences), healthcare professionals, and policy makers. This ensures a comprehensive approach to the development of a recruitment strategy.

The study will be conducted in Ireland, a country with a healthcare system that includes both public and private sectors. The government body responsible for overseeing the public health system is the Health Service Executive (HSE), and approximately 47% of Irish citizens are covered by private health insurance. However, in May 2021, the Irish government approved the Sláintecare Implementation Strategy and Action Plan 2021 to 2023, aiming to transform this dual system to a universal healthcare structure. The National Cancer Control Programme (NCCP) is the HSE's cancer-specific division, accountable for the organisation and governance of publicly-funded cancer services in the country.

Ireland is currently grappling with significant lung cancer morbidity and mortality. Lung cancer is the fourth most common cancer, with roughly 2,749 people diagnosed each year as of 2020 ^42^. Mortality rates remain high, largely due to late-stage diagnosis. The potential benefits of lung cancer screening have been recognised at the national level. Advocacy, charitable organisations, and public representatives have proposed the introduction of a community-based lung cancer screening program as a proactive approach to address the country's lung cancer burden. In preparation for this screening program, a robust recruitment strategy is needed to address inequalities that may arise from inequitable participation.

### Target population

This study is being conducted alongside an ongoing pilot clinical trial of lung cancer screening: the LHC trial ^43^. The LHC trial is being deployed in communities within a relatively deprived Irish region with higher lung cancer incidence: North Dublin and the North East. Individual deprivation will also be assessed by educational attainment and employment status. This will ensure that individuals from deprived areas, defined as Electoral Divisions (EDs) categorised as “Disadvantaged” to “Extremely Disadvantaged” by the Pobal HP Deprivation Index, 2022 ^44^, will be predominantly, although not exclusively, included. This population is particularly important to target for lung cancer screening due to their elevated risk and increased vulnerability. These individuals are often underrepresented in healthcare research and are less likely to participate in screening programs, despite being at the highest risk of adverse outcomes. By focusing on this population, our study aims to design a recruitment strategy that is tailored to their specific needs and experiences, thereby improving their engagement in future lung cancer screening programs in Ireland. 

While our target population criteria are primarily based on age, smoking status, and socioeconomic factors, we are aware of the potential for cultural diversity within this group. Therefore, our recruitment strategy and subsequent intervention will be designed with cultural sensitivity in mind, ensuring that we respect and address the unique needs and experiences of diverse cultural groups within our target population.

### Study design

The intervention development will follow a four-step process:

1. **Establishing the evidence base**: An umbrella review of previous research on screening participation interventions will form the evidence base ^45^. The findings from this umbrella review, alongside other relevant studies will be presented to the stakeholder panel for feedback, which will be used to develop an initial version of the recruitment tool.
2. **Identification of Barriers and Facilitators**: We will conduct in-depth interviews with a sample of the target population to gain insights into their experiences and perspectives, thereby informing the development of the recruitment strategy.
3. **Modelling intervention processes and outcomes**: We will map the themes identified in steps 1 and 2 to the Capability-Opportunity-Motivation framework within the Behaviour Change Wheel (BCW) to draft a cohesive recruitment strategy ^35^. The research team and expert stakeholders will conceptualise the behaviour change mechanism and identify measurable processes and outcomes.
4. **Feasibility and acceptability**: We will present the initial draft of the recruitment tool to stakeholders and the PPI panel for review and refinement, assessing its feasibility and acceptability.

**Figure 1.**
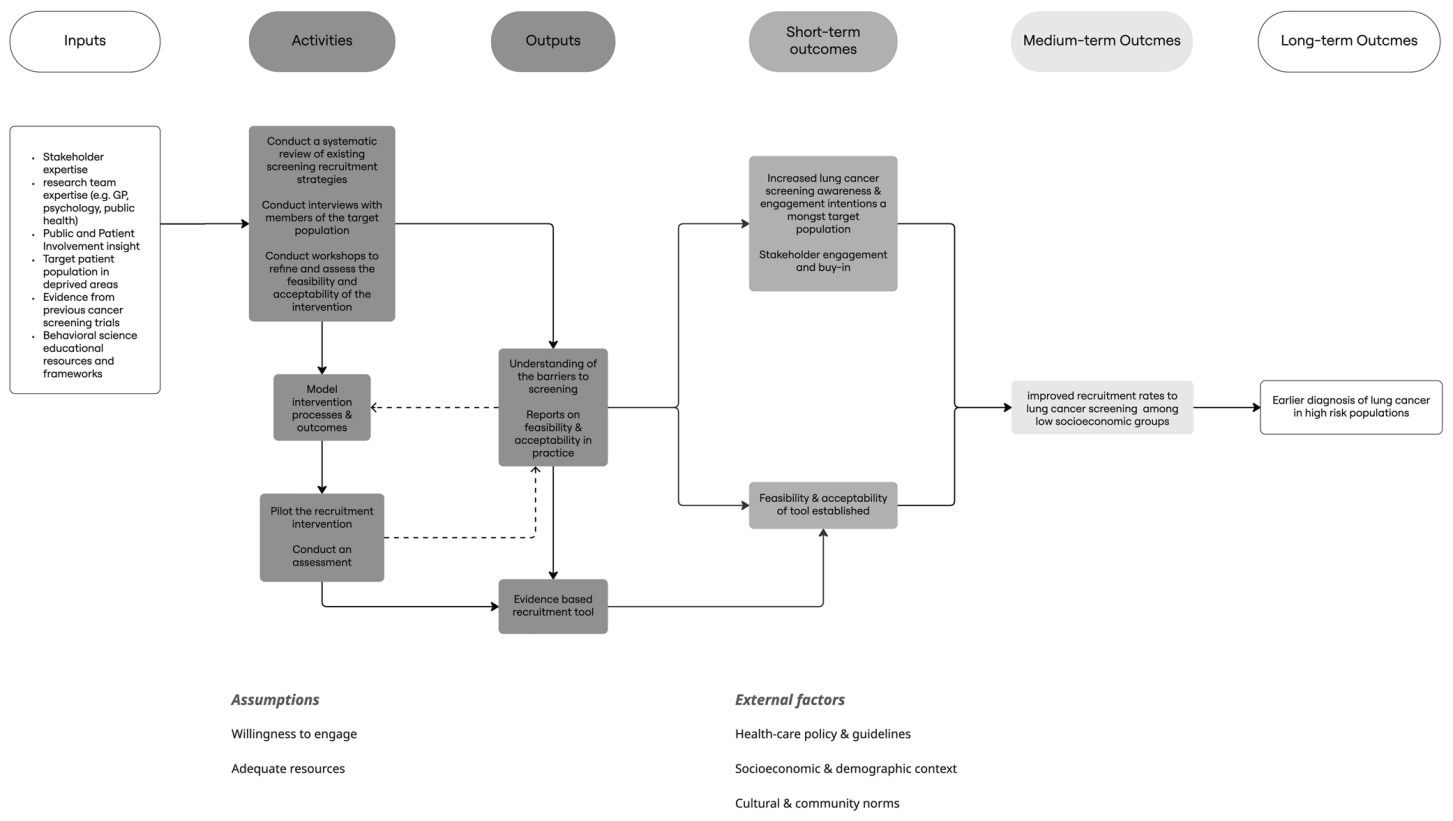
Logic model of proposed intervention.

The study outline is described in Figure 1 below.

### Sampling, data collection & analysis

The target population will be recruited from primary care practices and community centers involved in the ongoing LHC trial, which is being delivered in socioeconomically deprived regions of North Dublin and the North East. Eligibility is based on the NLST criteria: participants must be aged 55–74 with a smoking history of at least 30 pack-years. Former smokers must have quit within the past 15 years. Individuals enrolled in our ongoing LHC trial are also eligible, given the trial's inclusion criteria – aged 55–74 with either a PLCOm2012 risk score above 1.5% or an LLPv3 score of 2.5% or higher. Exclusion criteria include those with a current malignancy, lung pathology, life-limiting illness, or inability to provide informed consent in English. While recruitment will primarily focus on high-risk individuals from disadvantaged communities, it will not be limited exclusively to them. We will collect demographic information including age, gender, smoking history, and socioeconomic status, assessed using the Trinity National Deprivation Index (2016) ^46^ education level, and employment status.

The audio files of interviews will be de-identified and transcribed. NVivo 10 or higher (QSR international) will be used for analysis. For analytical rigour, 40% of the transcripts will be independently double coded by another researcher. We will use the Consolidated Framework for Implementation Research (CFIR) to classify the themes that emerge ^47^.

Workshops involving stakeholders, the PPI panel, and healthcare professionals will form the final evidence base to evaluate and refine the feasibility and acceptability of the recruitment strategy. The discussions during the workshops will be audio-recorded and supplemented with field notes taken by the research team.

### PPI

The PPI group will include individuals and/or families with experience of lung cancer, as well as members of the target lung screening population. Some members may be affiliated with the ILCC. Mr Cotter from the ILCC will support the formation of a small panel (4–5 members) and act as a liaison with the research team to coordinate meetings. The PPI panel will provide input on the interview topic guide and offer feedback during the intervention development process. 

***Interview topic guide design***

Developing a topic guide based on behavioural science frameworks will be a collaborative endeavour. The PPI group will engage in an initial brainstorming workshop, followed by a series of feedback sessions, and they will also be invited to provide written input to refine the guide.

***Training and support***

All PPI members, regardless of their background, will be offered training on the research methodology, ethics, and behavioural science principles. This is to ensure that everyone is adequately equipped to provide meaningful insights and feedback throughout the project's lifecycle. Regular support, both technical and psychological, will be available to facilitate their active participation.

***Schedule of meetings***

A proposed series of meetings will include:

**Introduction session:** Overview, objectives, and roles.

**Topic guide workshop:** Brainstorming and initial draft creation.

**Bi-monthly collaborative meetings:** Progress updates, discussions, and feedback sessions.

**Final review meeting:** Reflecting on results and laying out the dissemination roadmap.

***Oversight and dissemination***

All PPI members will be encouraged to actively partake in the research dissemination process. Opportunities to co-author research outputs, present findings at conferences, and engage in outreach activities will be provided. This inclusive strategy has been shaped with valuable guidance from the RCSI PPI Ignite Office Team.

### Expert panel

An expert panel consisting of primary care clinicians, psychologists, and policymakers will be convened to contribute specific expertise to this project. Each panel member has been selected based on their roles and experiences in the context of lung cancer screening. They bring critical insights that can significantly influence the future implementation of the lung cancer screening trial.

The expert panel will be actively involved in various stages of the study. They will provide feedback on the evidence gathered from systematic reviews, participate in workshops to discuss and refine the recruitment strategy, and contribute to the design of the recruitment tool based on their professional experience and understanding of the challenges and opportunities in lung cancer screening. Furthermore, they will provide critical insight into the likely barriers and facilitators for implementing the recruitment strategy in a real-world context.

To ensure a diverse range of perspectives, the Expert Panel is drawn from various sources. These include professional networks with a wealth of knowledge and experience, representative organisations that provide a broad societal perspective, and patient advocacy groups that offer the unique and essential viewpoint of those most directly affected by lung cancer.

### Step 1: Evidence gathering

The initial phase of our research will involve an extensive review of existing literature including systematic reviews, observational studies, and qualitative research on interventions aimed at promoting participation in screening programs within general practice. This study follows a comprehensive synthesis of systematic reviews conducted earlier by our team ^45^. The outcomes of these reviews will be shared in a meeting with key stakeholders. Factors which the review has identified as positively influencing participation in screening will be presented to the stakeholders. Stakeholder feedback will be considered, and a decision regarding which factors should be included in the initial iteration of our recruitment tool will be reached via consensus using a modified Nominal Group Technique, which gives an equal voice to all participants ^48, 49^

### Step 2: Identification of barriers and facilitators

We will conduct a series of in-depth semi-structured interviews with a representative sample of our target population to gauge their attitudes and beliefs towards lung cancer, as well as their likelihood to participate in a screening program. These interviews will consist of standardised, open-ended questions and will be audio-recorded and transcribed with the interviewees’ written informed consent. The interviews will be semi-structured to facilitate the participants giving a full account of their health behaviours in relation to smoking and cancer screening. Demographic data including age, sex, education, employment status and post-code, as well as smoking data (self-reported smoking status, smoking history and maximum number of cigarettes smoked per day). Subsequently, they will be subjected to thematic analysis. Our objective is not to be confined to a predetermined number but to continue interviews until saturation, where no new themes emerge. However, based on our initial estimates, we anticipate engaging with approximately 30 individuals. We will recruit participants from primary care practices and community centers, screen them based on specific inclusion criteria. Eligibility is based on the LHC trial and the NLST criteria. 

### Step 3: Intervention modelling

Behavioural science frameworks such as the Behaviour Change Wheel (BCW), the Extended Parallel Processing Model, and the Theoretical Domains Framework (TDF) ^35, 43, 50^ will underpin our recruitment strategy. The patterns identified in the first two steps will be aligned with the Capability-Opportunity-Motivation framework within the BCW, and a coherent recruitment intervention strategy will be drafted ^35^. A consultation will be held with our expert stakeholders to conceptualise the mechanism of behavioural change and identify behavioural targets, along with measurable processes and outcomes.

### Step 4: Feasibility and acceptability

Once we have an initial draft of the recruitment tool, it will be presented to our key stakeholders and PPI panel in workshops for review. The tool will be refined based on their feedback regarding the acceptability and usability of individual intervention components. This cycle of presentation, feedback, and refinement will continue until we attain agreement on the final intervention package.

***Workshops***

In the workshops, we will facilitate monitored discussions in small groups, each comprising no more than five members from our expert stakeholder panel or PPI group. A research team member will guide each group discussion, supplying prompts to stimulate dialogue.

### Purpose of workshops

The workshops will serve as the final stage in consolidating our evidence base, allowing us to evaluate and refine the recruitment strategy's feasibility and acceptability. We will invite participants to one of four workshops, where they will review logic models prepared by the research team. In the style of World Café methodology, participants will reflect on the recruitment intervention's components and how these integrate within existing care pathways ^51^. These workshops will also enable us to combine the personal narratives of patients with the clinical insights from our stakeholders. For patients, we will explore factors like burden of care, guideline appropriateness, preferences, and support types. The recurrent themes from these discussions will inform the project's feasibility. We will report our findings using the COREQ framework, ensuring comprehensive and accessible data ^52^. The output will be the blueprint for the architecture and content of the final recruitment tool.

### Workshop structure

In the workshops, we will organise small table group discussions. Each table will randomly seat no more than five members from the expert stakeholder panel or PPI group, with members rotating between tables for different discussions. A research team member will be present at each table during the World Café-style workshop to provide discussion prompts. Each table will concurrently discuss the same prompts over three rounds, with each round featuring a new prompt.

At the start of each session, we will present a PowerPoint (accompanied by handouts) summarizing findings from Step1–3. Subsequent workshops will begin with brief presentations recapping insights from previous sessions. While the workshops will not undergo pilot testing, they will follow a format similar to that used in previous health intervention co-design processes ^39, 53^. The prompts given for discussion will be mapped to the APEASE criteria to ensure that the implementation of the intervention is considered at every stage of the iterative co-design process ^36^. We will hold four workshops in total, with each one building on the insights gleaned from the preceding workshop and featuring unique discussion prompts.

### Workshop data collection and analysis

With the consent of all participants, we will audio record the discussions. The research team will also take field notes during the discussions. We anticipate that each workshop will last approximately one hour. Data saturation will be determined via consensus among the research team members, who will continue table discussions until no new themes emerge.

***Initial pilot***

Following the workshops, a preliminary pilot will be embedded in the regional lung health check programme. The proportion of participants who receive the recruitment intervention and subsequently consent to participate in the trial recorded for the duration of the pilot.

A reporting system will be established to ensure that key implementers can report any problems with the dissemination, reception or response to the recruitment tool, or any problems with the transition from positive response to the invitation to the LDCT. Participants will have the opportunity to provide feedback on their experience of being recruited via anonymous survey which will be available at their participating GP practice. The pilot test will aim to assess the feasibility of the tool - how well it operates in a real-world setting - as well as its acceptability to patients, which is crucial for its eventual uptake. Feedback collected from the participants in this phase will be used to further optimise the tool and ensure it is both practical and user-friendly before larger scale piloting and ultimately definitive testing.

***Intervention description***

At the conclusion of the intervention development process, the final intervention components will be reported according to the TIDieR guidelines to ensure completeness and replicability ^54^. We will describe the rationale and theoretical basis for the intervention in detail. We will also list the materials that are required to deliver the intervention and the training materials that are required to train staff in its use.

***Study status***

Our present study status indicates that we have duly submitted our ethics application and are presently awaiting their response.

## Discussion

This study represents a significant step forward in the development of an effective recruitment strategy for lung cancer screening in high-risk individuals. By using a collaborative, evidence-based approach and incorporating insights from patients, public involvement contributors, and expert stakeholders, we hope to enhance the reach and impact of lung cancer screening programs.

The recruitment strategy under development has the potential to inform the design of future lung cancer screening programs, not just in Ireland, but also in other countries that may face similar challenges in reaching high-risk populations. Additionally, the evidence gathered in the course of creating this recruitment strategy will add crucial information to a currently limited evidence base on lung cancer screening in Ireland.

### Dissemination

The findings of this study will be disseminated through multiple channels. These include academic journals, presentations at national and international conferences, and reports to relevant health and governmental organisations. In particular, we aim to communicate our findings to the public and to the communities most affected by lung cancer.

### Limitations

There are limitations to this study. We use the Pobal National Deprivation Index to screen members of the target population for low economic status because it is a standardised measure of deprivation specifically created for public health research ^44^. However, the most recent update of this index contains data from 2022, which may be out of date. It also may not accurately reflect the individual economic status of each recruited member. We have attempted to mitigate these limitations by including questions about the participants’ highest educational attainment and employment status in the interview.

Additionally, much of the existing evidence on recruitment to lung cancer screening that will be reviewed in step 1 to inform the creation of our recruitment strategy is from countries that have conducted lung cancer screening trials. Some of the qualitative research regarding the beliefs and behaviour of smokers may not be directly applicable to our target population due to cultural differences. The in-depth interviews with our target population described in step (2) localise the qualitative evidence and contribute to lung cancer screening recruitment evidence in Ireland.

Furthermore, we are also exploring digital methods in parallel work and hypothesize that a hybrid approach, combining primary care recruitment methods with digital self-referral or outreach techniques, may yield the best results in increasing lung cancer screening participation in Ireland. The tool co-developed in this study is designed to meet the specific needs of Ireland’s high-risk populations, particularly those in socioeconomically deprived areas, where a majority are eligible for publicly funded healthcare and are registered with a GP practice ^55^.

Finally, it is important to acknowledge that prioritizing screening for individuals at highest risk, such as heavy smokers, may be constrained by the prevalence of impaired lung function within this group, which is associated with the development of more aggressive lung cancers and poorer prognoses ^56^. While this limitation may attenuate the clinical benefits of early detection, directing screening efforts toward high-risk individuals, especially those from socioeconomically deprived areas, aligns with broader public health objectives aimed at enhancing equity in access to preventive care. Consequently, notwithstanding variability in clinical outcomes, expanding access to screening represents a critical public health imperative.

## Conclusion

This protocol outlines the plan for the co-design of a lung cancer screening recruitment strategy. The outcome of this intervention will enable specific recommendations to be made regarding recruiting participants to maximise the efficacy of a future all-Ireland lung cancer screening programme. Such a programme may give due consideration to targeting groups which are historically underrepresented in health intervention services including screening programmes.

## Data Availability

No data are associated with this article. Open Science Framework: Co-designing a recruitment strategy for lung cancer screening in high-risk individuals: protocol for a mixed-methods study, https://doi.org/10.17605/OSF.IO/W4KMT ^57^. This project contains the following extended data: Participant consent form PPI Interview Participant Information Leaflet Interview guide Workshop plans Pilot Survey (Anonymous) Data are available under the terms of the Creative Commons Zero "No rights reserved" data waiver (CC0 1.0 Public domain dedication).
