# Supplementary material for: Co-designing a recruitment strategy for lung cancer screening in high-risk individuals: protocol for a mixed-methods study
Source: HRB Open Res. 2025 Jul 28;6:64. Originally published 2023 Nov 13. [Version 2] doi: 10.12688/hrbopenres.13793.2 (PMC11190654; doi:10.12688/hrbopenres.13793.2)
Supplement: Peer review history

# Reviewer report: Reviewer response for version 2

**DOI:** 10.21956/hrbopenres.15635.r48594

**Published:** 2025-08-20

**Authors:** Gaston Arnolda

**Affiliations:** 1 Macquarie University, Sydney, New South Wales, Australia

I have read the authors' response to my comments on the first draft. I can confirm that the clarifications I required were addressed in their responses, with appropriate modification to the manuscript. 

 I particularly appreciated the updated logic model. If it is possible, it would be helpful if the pdf can place this logic model on a full landscape-formatted page so that it can be viewed simply when the pdf is printed out.

 My initial assessment was 'Approved' and I see no reason to downgrade this.

Is the study design appropriate for the research question?

Partly

Is the rationale for, and objectives of, the study clearly described?

No

Are sufficient details of the methods provided to allow replication by others?

No

Are the datasets clearly presented in a useable and accessible format?

Not applicable

Reviewer Expertise:

NA

I confirm that I have read this submission and believe that I have an appropriate level of expertise to confirm that it is of an acceptable scientific standard.

# Reviewer report: Reviewer response for version 1

**DOI:** 10.21956/hrbopenres.15091.r39808

**Published:** 2024-06-20

**Authors:** Abbie Begnaud

**Affiliations:** 1 University of Minnesota Medical School, Minneapolis, USA

This protocol is an important contribution to the field of advancing lung cancer screening uptake in Ireland. Much of the published work has occurred elsewhere and thus, needs to be adapted to the local context. Community co-design is a valid and ideal approach, integrating evidence based strategies with behavioral health frameworks and lived experience of the population of interest. Not knowing the specific guidelines or coverage for lung cancer screening in Ireland, I’m not sure if the selected population (persons aged 55 and older who have recently quit smoking or are still smoking cigarettes) is appropriate. Why not include individuals aged 50 and older or those who have quit longer ago? I also did not see mention of incentives for participants (PPI or interview participants) and expect these will be offered? Finally, it’s not clear to me what “Irish Lung Cancer Community (ILCC)” is - is this a general description of people in Ireland personally affected by lung cancer or is this a specific named group or organization?

Is the study design appropriate for the research question?

Yes

Is the rationale for, and objectives of, the study clearly described?

Yes

Are sufficient details of the methods provided to allow replication by others?

Yes

Are the datasets clearly presented in a useable and accessible format?

Not applicable

Reviewer Expertise:

Lung cancer screening and implementation research

I confirm that I have read this submission and believe that I have an appropriate level of expertise to confirm that it is of an acceptable scientific standard.

# Reviewer report: Reviewer response for version 1

**DOI:** 10.21956/hrbopenres.15091.r39809

**Published:** 2024-06-03

**Authors:** Mario Mascalchi

**Affiliations:** 1 "Mario Serio", University of Florence, Florence, Italy

The project is thoroughly and redundantly described.

 It aims to define a strategy suited to improve participation to Lung Cancer Screening in smokers living in deprived areas of Ireland.

 Merits

 The process to define an optimal recruitment strategy for the target population is well analyzed and subdivided in 4 steps with involvement of different components (clinical scientists, stakeholders etc.)

 Limitations

 The assumption that recruiting subjects at higher risk of LC focusing in deprived areas will yield a major benefit in term of LC screening efficacy, is at odds with the observation that, unfortunately, subjects at higher risk based on age and smoking burden are those that more likely develop more aggressive and untreatable LC (Young et al, 2022) [Ref 1]

 Starting from GP is not the unique approach to identify eligible subjects. In Europe, self-referral through web is feasible and brings to recruitment of subjects with less smoking co-morbidities, primarily cardio-vascular disease and chronic obstructive pulmonary disease, which often vanish the potential benefit of a LC discovery through screening. I understand that subjects in deprived areas of Ireland are not likely to use the web, but a smartphone is probably owned by every 55-70 years old subject living in Ireland.

 The Authors should provide some clearer hypotheses about which recruitment modality or mix of factors involved in recruitment modality are expected to constitute the outcome of the project.

Is the study design appropriate for the research question?

Partly

Is the rationale for, and objectives of, the study clearly described?

Yes

Are sufficient details of the methods provided to allow replication by others?

Partly

Are the datasets clearly presented in a useable and accessible format?

Partly

Reviewer Expertise:

NA

I confirm that I have read this submission and believe that I have an appropriate level of expertise to confirm that it is of an acceptable scientific standard, however I have significant reservations, as outlined above.

## References

[1] : Lung function impairment in lung cancer screening: discordance between risk and screening outcomes when looking through a PRISm. *Translational Lung Cancer Research* .2022;11(10) : 10.21037/tlcr-22-634 1988-1994 10.21037/tlcr-22-634 PMC964103936386460

# Response

**Published:** 2024-10-22

**Authors:** Sam McGlynn

**Affiliations:** Royal College of Surgeons in Ireland Department of General Practice, Dublin, Leinster, Ireland

Hi Mario,

 Thank you for taking the time to read our article and for the detailed comments provided. We have attempted to address these below and hope that they will ease any reservations you had previously.

 You raise a valid point regarding the potential benefit of screening in terms of early detection and treatment efficacy being diminished in high-risk populations due to the development of more aggressive forms of lung cancer. We will look at adding a new section to Limitations heading in the protocol to acknowledge this issue and reference Young et al. (2022). However, the rationale for focusing on deprived areas is to increase equity in access to screening and enhance uptake. It therefore aligns with the broader goals of public health, even if the clinical benefits may vary depending on the aggressiveness of the detected cancers. 

 Your second comment relates to adding self-referral through web-based platforms or smartphone apps and this could be a useful addition. We will add an approach in the Methods section that outlines the potential of incorporating digital recruitment methods alongside recruitment through GPs. There are potential barriers to digital access in deprived areas but this could potentially help reach a wider audience and improve participation rates. 

 Finally, we will add specific hypotheses regarding the recruitment modalities and combinations we expect to be most effective, that tie in with the above comment. “We hypothesise that a hybrid recruitment strategy—combining traditional general practice recruitment with digital self-referral methods—will yield higher participation rates compared to a single-modality approach.” In addition to this we could look at adding a framework for evaluating the effectiveness of different recruitment strategies based on metrics such as recruitment rate, participant diversity, and participant engagement.

# Reviewer report: Reviewer response for version 1

**DOI:** 10.21956/hrbopenres.15091.r37037

**Published:** 2023-11-30

**Authors:** Gaston Arnolda

**Affiliations:** 1 Macquarie University, Sydney, New South Wales, Australia

Thank you for this protocol outlining a process of co-designing a recruitment strategy for people at higher-risk of lung cancer. Multiple methods are proposed to devise the strategy in four steps: 1) systematic review of interventions; 2) interviews with the population targeted for screening; 3) development of a strategy; and 4) assessment of feasibility and acceptability. People at higher risk are the targets in step 2, while consumer representatives with a focus on lung cancer will play a role in step 4 (and perhaps step 1 - not entirely clear). A separate expert group comprising primary care clinicians, psychologists and policy makers will be involved in steps 1-4, in various ways. 

 The purpose of co-developing a recruitment strategy is generally recognised as best practice and all of the proposed processes are in themselves not particularly controversial. I am unclear about many details, however, and thus unable to seek specific clarification at this point in the review process as the aim and initial logic model is not entirely clear to me (please accept my apologies for any misunderstandings on my part), and I am also unclear on what is planned in some of the steps. I will do my best to outline the main issues that I believe would be helpful to clarify in a revision.

Aim: The aim as specified in the abstract and the end of the introduction is stated as developing a strategy for recruiting older people at higher-risk of lung cancer (particularly in deprived populations). In the methods, however, the sampling for the target group is restricted to eligible low SES individuals attending (an unspecified number of) general practices. This creates crucial questions for me. If the aim is to target eligible low SES people in general, it is not clear to me that this is an appropriate sampling strategy for step 2; this would need to be justified (i.e., evidence that they all  regularly attend GPs). If on the other hand the strategy is explicitly to intervene in relation to recruitment through general practices this should be stated in both the aims and and at the end of the introduction and, while the sampling of GP patients would be appropriate, the question arises as to why general practice staff are not a separate group for interview and participants in co-development of strategies (outside of the 'expert group').

Logic model: While I appreciate that the logic model is rudimentary at this stage, it would help the reader if it was re-framed to show how inputs and activities contribute to outputs and short- and longer-term outcomes; the current graphic does not make this clear. A recent paper on the subject may be helpful (Mills, T., Lawton, R. and Sheard, L., 2019. Advancing complexity science in healthcare research: the logic of logic models. BMC medical research methodology, 19(1), pp.1-11). As a trivial aside, the finalised logic model needs to have a higher optical resolution.

'High-risk' target group: I found the term high-risk distracting, as it suggests a risk threshold above which someone will be a target. My reading of your protocol, however, is that you are targeting eligible lower SES individuals in lower SES settings, regardless of their individual risk. If this is correct, I suggest reframing the terminology to refer to recruiting eligible low SES individuals in low SES communities (who are higher risk of lung cancer by virtue of this SES).

Step 1 sytematic review(?s): It is not clear to me whether the study is proposing one or more systematic reviews. If one review only, the protocol needs to justify why the already published review of interventions to promote participation in screening programs (Ref #19) was the only information required (e.g., no other information is required vs other information is required, but is adequately provided by other up to date reviews). The information needs should be depicted in the logic model.

Step 2 interviews: I may be misunderstanding this, but it seems to me inappropriately labelled 'theory formulation', which I find confusing. Rather, it seems to me that the purpose is to interview the target population to identify potential barriers and facilitators to participating in screening. Having identified these barriers and facilitators, there may then be a process, drawing on theory, to identify a subset of candidate interventions (which seems to me to be Step 3). If you agree, the title 'Identification of barriers and facilitators' seems ore appropriate.

 Thanks for the opportunity to comment on what I trust will be an important piece of research.

Is the study design appropriate for the research question?

Partly

Is the rationale for, and objectives of, the study clearly described?

No

Are sufficient details of the methods provided to allow replication by others?

No

Are the datasets clearly presented in a useable and accessible format?

Not applicable

Reviewer Expertise:

Health Services Research

I confirm that I have read this submission and believe that I have an appropriate level of expertise to confirm that it is of an acceptable scientific standard, however I have significant reservations, as outlined above.

# Response

**Published:** 2024-10-22

**Authors:** Sam McGlynn

**Affiliations:** Royal College of Surgeons in Ireland Department of General Practice, Dublin, Leinster, Ireland

Hi Gaston,

 Thank you for taking the time to read our article and for your detailed comments. We have attempted to address these below and hope that they will ease any reservations you may have had previously.

 We will be revising the aims of the study to clearly indicate that the recruitment strategy is specifically focused on low SES individuals attending general practices in deprived areas. We will also be updating the methods section to include a plan for conducting separate focus groups with general practice staff to gather their perspectives on the recruitment strategy. 

 We will aim to more clearly depict the relationship between inputs, activities, and outputs, as well as short- and long-term outcomes in the logic model. The finalised graphic will demonstrate that the logic model reflects the complexity of the intervention and provides a transparent overview of the research process as in Mills et al. (2019).

 As suggested, we shall change terminology throughout from “high risk” to “eligible low SES individuals in low SES communities” to better reflect the study’s aims.

 To clarify, only one systematic review will be conducted to gather evidence from existing interventions to promote participation in screening programs, supplemented by additional observational studies if necessary. This should provide comprehensive coverage of relevant literature and is in alignment with our research objectives.

 Finally, we will revise the title of Step 2 to ‘Identification of Barriers and Facilitators’ to better capture its intent, which involves conducting interviews to understand the target population’s perspectives on screening participation.
